# Pandemic (H1N1) 2009 Influenza Community Transmission Was Established in One Australian State When the Virus Was First Identified in North America

**DOI:** 10.1371/journal.pone.0011341

**Published:** 2010-06-28

**Authors:** Heath A. Kelly, Geoff N. Mercer, James E. Fielding, Gary K. Dowse, Kathryn Glass, Dale Carcione, Kristina A. Grant, Paul V. Effler, Rosemary A. Lester

**Affiliations:** 1 Victorian Infectious Diseases Reference Laboratory, Melbourne, Australia; 2 National Centre for Epidemiology and Population Health, Australian National University, Canberra, Australia; 3 Department of Health, Melbourne, Australia; 4 Department of Health Western Australia, Perth, Australia; University of Swansea, United Kingdom

## Abstract

**Background:**

In mid-June 2009 the State of Victoria in Australia appeared to have the highest notification rate of pandemic (H1N1) 2009 influenza in the world. We hypothesise that this was because community transmission of pandemic influenza was already well established in Victoria at the time testing for the novel virus commenced. In contrast, this was not true for the pandemic in other parts of Australia, including Western Australia (WA).

**Methods:**

We used data from detailed case follow-up of patients with confirmed infection in Victoria and WA to demonstrate the difference in the pandemic curve in two Australian states on opposite sides of the continent. We modelled the pandemic in both states, using a susceptible-infected-removed model with Bayesian inference accounting for imported cases.

**Results:**

Epidemic transmission occurred earlier in Victoria and later in WA. Only 5% of the first 100 Victorian cases were not locally acquired and three of these were brothers in one family. By contrast, 53% of the first 102 cases in WA were associated with importation from Victoria. Using plausible model input data, estimation of the effective reproductive number for the Victorian epidemic required us to invoke an earlier date for commencement of transmission to explain the observed data. This was not required in modelling the epidemic in WA.

**Conclusion:**

Strong circumstantial evidence, supported by modelling, suggests community transmission of pandemic influenza was well established in Victoria, but not in WA, at the time testing for the novel virus commenced in Australia. The virus is likely to have entered Victoria and already become established around the time it was first identified in the US and Mexico.

## Introduction

The first confirmed case in Australia of pandemic (H1N1) 2009 influenza (pH1N1) was recorded in Queensland in a returned traveller from the United States (US) on 9 May 2009 [1], almost four weeks after the first cases were confirmed in the US on 15 and 17 April 2009 [2]. Victoria confirmed its first case in a traveller from the US on 20 May [3] and the first case in Western Australia (WA) was notified on 24 May in a traveller returned from Canada via the US [4].

The response to the identification of cases of pandemic influenza initially followed the guidelines of the Australian Health Management Plan for Pandemic Influenza (AHMPPI). [3], [5], [6], Management of the pandemic moved through three phases, described as *Delay*, *Contain*, and *Protect*. An additional phase, *Modified Sustain* was applied only in Victoria. The phases were designed to *delay* the entry of pandemic virus into Australia, to *contain* the virus once it had entered the country, to *sustain* a response once community transmission had been established and to *protect* the vulnerable once infection was deemed to be widespread [5], [7].

*Modified Sustain* was announced in Victoria on 3 June 2009 – just more than two weeks after the first confirmed case in Victoria – and was followed by a decrease in active case finding. Two weeks later, on 17 June, the Australian Government announced the *Protect* phase, one that had not been included in Australia's pandemic plan, but was added to the original AHMPPI[5], [7] once the less severe nature of the pandemic had been accepted. WA pre-empted formal adoption of the *Protect* phase on 13 June, when all doctors and hospitals were asked to cease active case-finding, and prioritise influenza testing only to persons with severe influenza-like illness or established medical risk conditions. Victoria formally moved to the *Protect* phase on 23 June.

Although the first laboratory-confirmed cases were identified in Victoria and WA within four days of each other, reported case numbers immediately escalated in Victoria but not in WA [8]. We suggest this observation is explained by the unrecognised establishment of community transmission of pH1N1 in Victoria, but not in WA, around or before 26 April, when public health agencies and laboratories in all Australian states and territories (“jurisdictions”) commenced investigating and testing incoming travellers with influenza-like illnesses from North America for pandemic influenza. We support our argument with a detailed review of case follow-up data for both states, a review of other surveillance data relevant to Victoria and modelling of the epidemic in both states.

## Methods

### Background

We compared Victoria and WA because they do not share a border and a large distance separates these two states, allowing for importation between states to be more readily recognised. The state capitals, Melbourne and Perth, are approximately 3,400 km apart by road, on the south-east and west coasts respectively. Victoria has a population of approximately 5.4 million of whom 70% live in Melbourne, while the WA population is estimated as 2.2 million, with 74% living in Perth.

As part of the *Delay* and *Contain* phases of the Australian response to the pandemic, active case-finding involved identification, isolation, testing and antiviral treatment of incoming travellers with influenza-like illnesses; and prophylactic treatment and home quarantine of the close contacts of suspect/confirmed cases. Influenza is a notifiable disease in all Australian jurisdictions. Public health reference laboratories in Victoria and WA developed pH1N1-specific nucleic acid amplification tests in the first week of May.

Early spread of the pandemic virus in both states was concentrated in the capital cities [3]. High quality case ascertainment and contact follow-up data were available from both states. Of all Australian jurisdictions, community transmission of pandemic influenza was established earliest in Victoria and latest in WA.

### Case ascertainment and follow-up

Case ascertainment and follow up has been described in detail for Victoria [3]. Until 3 June, when the *Modified Sustain* phase was implemented, an attempt was made to identify and confirm every case and to follow-up every contact of suspected or confirmed cases. Until this date, 977 cases were identified and 5,807 contacts were followed-up. In WA prior to the formal implementation of the *Protect* phase on 13 June, all suspected or confirmed cases were actively followed up and travel histories were recorded. By this date, 102 cases had been confirmed and 232 household contacts of these cases followed-up, plus a large number of other contacts, including those on aeroplanes and at schools.

Other relevant data were gathered from international outbreak reports, postings on the electronic noticeboard ProMED-mail (http://www.promedmail.org) and a range of other electronic media reports.

### Calculation of the effective reproduction number for influenza H1N1 2009

The basic reproduction number (R_0_), indicates the average number of people each infected person infects in a totally susceptible population. By contrast, the time dependent effective reproduction number (R), indicates the average number of people each infected person infects, given the current interventions in place, and any prior immunity that reduces the susceptible pool. The effective reproduction number is always less than or equal to the basic reproduction number and typically declines gradually as a disease spreads through the population and collective immunity increases. The effective reproduction number was calculated using an adaptation of the method of Bettencourt and co-workers [9]–[11] to allow for imported cases and a distributed serial interval. The adaptation consists of cases being partitioned into local (L) and imported (M) cases and these are tracked in the data so that the new imported cases are not considered to be locally acquired infections and hence are not attributed to infection from previous local cases.

This method uses a stochastic version of the standard SIR (susceptible, infective, recovered) model and Bayesian inference to determine a probability distribution for R that best matches the case report data.Let τ denote the time interval between case reports (taken to be daily here) and in the time interval (t−τ, t) the number of locally acquired cases is L(t) and the number of imported cases is M(t). Implicit in the adaptation used here is the assumption that the imported cases spend their infectious period in the jurisdiction of interest which is reasonable given those cases where reported in that jurisdiction. The model uses discrete Euler time step approximations to the derivatives and so the new imported cases can be added at each discrete time step without affecting the model. The usual SIR infective equation can be written to the same degree of accuracy as in [9]–[11] as
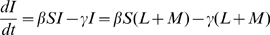
According to the SIR model, detailed in [9]–[11], the number of newly acquired local cases at time t+τ due to the L+M cases at time t is then given by:
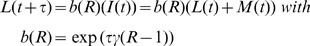
where γ is the mean infectious period. At each time step the new imported cases are added to obtain the total number of cases that give rise to locally acquired infections in the following time period.

Since daily case numbers are highly variable a probabilistic model is needed to allow for this variation. For a given R the probability of L local cases at time t+τ depends on the number of local and imported cases at time t and is given by:
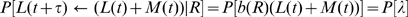
where *P*[λ] is a suitable probability distribution with mean λ. The difference between this and that presented in [9]–[11] is that the number of locally acquired cases is used as the data at time t+τ rather than all cases. The standard SIR model only deals with the average number of cases so a suitable probability distribution for *P*[λ] is a Poisson distribution which is the most general form (highest entropy) if only averages are known.

We are interested in estimating R and how it evolves over time as new cases are reported. That is, we want to know the probability distribution of R that best fits the available data. From Bayes theorem:
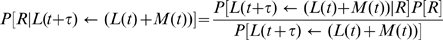
The denominator of the right hand side is simply a scaling factor that can be calculated from the sum of the probabilities being 1 and does not need to be explicitly determined. The first term in the numerator is calculated using the Poisson distribution, as discussed above, using the case numbers at time t+τ. *P[R]* is the *prior* probability distribution of R, which reflects earlier values of R, either from calculated values or initially from knowledge of the disease. Here an initial unbiased estimate of *P[R]* is chosen to be a uniform distribution on [0 4], that is any value of R in [0 4] is equally likely. The above equation is iterated to obtain progressively better estimates for the probability distribution of R as time progresses and more data become available. As a probability distribution for R is obtained, compared to other methods that produce a single value for R, a 95% credible interval for the R value is easily obtained.

We have verified this method using many thousand numerically simulated outbreaks with known values of R and different imported cases distributions. Over all different importation scenarios the method gave a reliable estimate of the underlying reproduction number. Previously, imported cases have either been removed from the calculations [12] or treated as local cases [13]. Both of these approaches overestimate the true effective reproduction number as either too much transmission is assigned to local cases or imported cases are assigned as being locally acquired, respectively.

For daily case report data considered here τ = 1, which is shorter than the serial interval of influenza. Using only the previous day's data (τ = 1), as outlined above, results in slow convergence of the method since changes in the case numbers are due mostly to the case numbers more than τ days earlier. Faster convergence and tighter bounds on R are obtained if R is calculated using a weighted sum of L and M stretching back in time beyond the serial interval. The weighting used is the temporal distribution of the serial interval, taken to be gamma distributed here, which weights earlier days relative to how likely the serial interval was to be that long. See below for further discussion on the serial intervals used in the analysis.

### Numerical simulation of outbreaks

We used mathematical models to calculate the number of days required for an outbreak initiated by a single imported case to reach the cases observed on 29 May in Victoria. The epidemiological parameters that most affect the growth rate of an outbreak are the reproduction number (defined above) and the serial interval. The serial interval measures the number of days between the time of infection of a secondary case and the time of infection of its infector. As discussed elsewhere [14], [15], both the distribution of the serial interval and its mean influence the growth rate of outbreaks. Following earlier work [12], [16]–[18] we have assumed a gamma distribution for the serial interval, and have considered a range of values for both the reproduction number and the mean serial interval.

Numerical simulations were performed in MATLAB using a stochastic version of a SEIR (susceptible, exposed, infective, recovered) type model. The code is available from the author (GM). A stochastic model was used rather than a deterministic SEIR model as it better reflects the variability inherent in the early stages of an outbreak. Inputs to the model were a fixed reproduction number (R) and a serial interval distribution (f(t), t = 1,…M) as described above. New cases at time t were sampled from a Poisson distribution with mean RS(t)∑f(τ)I(t−τ), where S(t) is the fraction of the population susceptible and I(t) are the number of infected individuals at time t. Initially, there were one million susceptible individuals and one infective case was introduced at time zero. We performed 1,000 simulations for each pair of values, and recorded the mean and standard deviation over these simulations. The simulations were not run beyond a total of 5,000 cases, so the results are insensitive to the initial population size, which was chosen large enough so that susceptible depletion was not an issue. Due to the stochastic nature of the method, not all simulations result in an established outbreak, with some resulting in what is known as stochastic die out [19]. Only those simulations that resulted in at least 20 cases were retained.

### Sensitivity to model parameters and assumptions

In order to obtain a robust estimate of the time taken for case numbers to reach those observed on 29 May, we considered values of the reproduction number ranging from 1.2 to 1.8 and mean serial interval from 2 to 4 days, consistent with other estimates of the mean serial interval of pandemic H1N1 of 1.9 days [20], 2.8 days [12], [16], [21], 3.2 days [22] and longer [23]. We performed 1000 simulations for each pair of values, and recorded the mean and standard deviation over these simulations.

While the serial interval and the reproduction number are the key factors that determine the speed at which an outbreak takes off, heterogeneities in contact patterns may also have some impact. One of the most likely sources of heterogeneity for this data arises from age structure [17]. The stochastic SEIR model described above is homogeneous with respect to age structure. This may be a limitation of the model. We therefore tested the impact of age heterogeneity using an alternative model with different reproduction numbers for adults and children (but with the overall reproduction number equal to that of our basic model). In order to test the impact of very high levels of heterogeneity, we assumed that the reproduction number for children was twice that of adults, due to heightened mixing between children and lack of prior immunity. The structured model estimated a reduction in 20–25% in the days required to reach the case numbers. In particular, for the intermediate case of a reproduction number of 1.4 and mean serial interval of 2.8 days, the delay was reduced from 33 to 26 days. The Victorian data could not be reproduced with this age-structured heterogeneity without using unrealistically large values of the reproduction number. The same was true of the stochastic SEIR model described above and other common simulation models such as deterministic SIR and SEIR type models [24]. We concluded that, although model structure influenced the estimate of the delay, even very high levels of heterogeneity had a relatively minor impact relative to the effect of the reproduction number or the serial interval, which are the dominant factors in determining the speed of the spread of the outbreak.

### Ethics statement

This research was exempted from ethical review under the Australian Government National Health & Medical Research Council's ‘National Statement on Ethical Conduct in Human Research’ because it was defined as negligible risk and involved the use of existing collections of data and records that contain only non-identifiable data about human beings. This study used aggregated notifiable diseases data that were collected under the relevant public health legislation in Victoria and WA.

## Results

### Pandemic cases in Victoria

**Figure 1:**
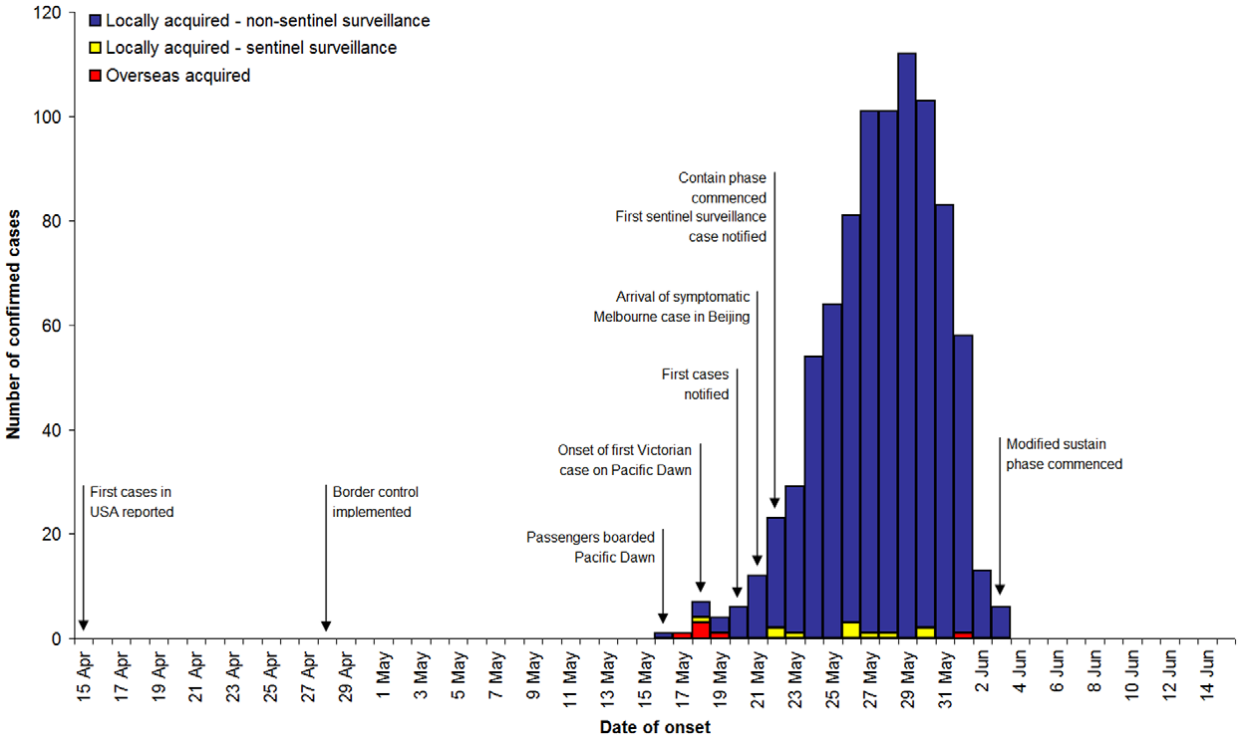
Notified cases by onset date and source and dates of significant events, Victoria 2009. All cases notified to the department until 4 June inclusive were included in the analysis as they were assumed to be tested during the Delay or Contain phases. Due to the delay between symptoms onset and notification, the number of cases in this chart decreases in the days prior to 4 June.

The first laboratory confirmed case in Victoria was notified on 20 May. Figure 1 shows notified cases by date of onset and location of acquisition until the commencement of the *Modified Sustain* phase; pandemic phase changes and case identification milestones are also indicated. Only 5% of the first 100 cases in Victoria were imported, and only eight of the 977 (0.8%) cases diagnosed prior to the introduction of the *Modified Sustain* phase reported a travel history. The first five diagnosed cases reported travel to the Americas: three brothers from one family returned from the US, a visitor from Mexico and another traveller returned from the US. All five cases were reported on 20–21 May. Two cases diagnosed on 1 June (numbers 368 and 374) were reported to have travelled to an affected country in the seven days prior to illness onset although the country was not specified for either case. One other case (number 398) diagnosed on 2 June, was reported to have acquired her infection in Japan.

### Surveillance evidence for early transmission in Victoria

An outbreak of influenza due to both pH1N1 and seasonal H3N2 influenza occurred on the cruise ship, the *Pacific Dawn* at a time prior to there being recognised transmission of pH1N1 in Australia. Of almost 3,000 passengers on the cruise, nine passengers with a Victorian residential address were subsequently confirmed to have pH1N1 infection. The earliest onset date of symptoms amongst the Victorian passengers was 18 May. Symptom onset for this patient occurred two days after boarding, consistent with a prodromal infection at the time of embarkation. Despite recognition of numerous passengers with influenza-like illness when the ship berthed in Sydney on 25 May, public health authorities allowed passengers to disperse into the community because the ship had not visited any port where there were confirmed cases of pandemic influenza. Retrospectively, it appears plausible that the Melbourne passenger who joined the cruise on 16 May 2009 with prodromal infection may have been the source of the shipboard pH1N1 outbreak.

Two other observations, which may also reflect a high point incidence of disease in Melbourne prior to recognition that local transmission was occurring. The eighth case to be diagnosed with pandemic influenza in Victoria was an eight-year-old male ascertained from routine general practice sentinel surveillance for influenza (Figure 1). This boy had no travel history or contact with travellers and symptom onset was on 18 May, two days prior to notification of the first confirmed Victorian case, the traveller from the US, who also had symptom onset on 18 May. Around that time in May, pandemic influenza was also exported from Melbourne to China. Amongst the first 12 cases in China, diagnosed between 11 and 25 May, one was a traveller from Melbourne who had arrived in Beijing on 21 May [25], only one day after Victoria's first case was confirmed.

### Estimation of the effective reproduction number for pH1N1 influenza in Victoria and simulation of the Victorian pandemic

**Figure 2:**
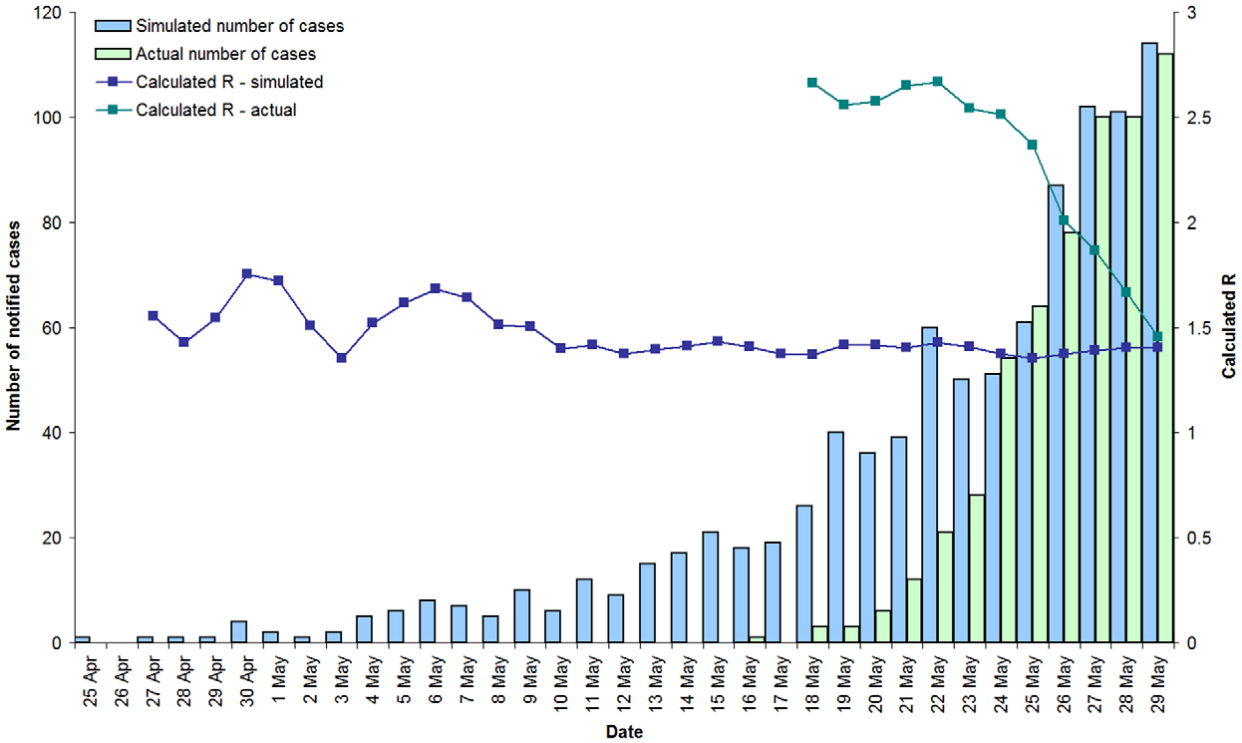
Actual and simulated cases and calculated R, Victoria, 25 April to 29 May 2009. An example of simulated data, calculated with an effective reproductive number (R) of 1.4 and a mean serial interval of 2.8 days, compared with observed data 25 April to 29 May, Victoria 2009. Also shown is R calculated from the observed data using a serial interval of 2.8 days.

Calculation of the reproductive number from the early Victorian data for all notified cases was performed as described in the methods section. Values ranged from R = 2.7 around 20 May, when the first Victorian case was reported, and fell steadily and dramatically to 1.5 by 29 May (Figure 2).

To test the hypothesis of early community transmission of pH1N1 in Victoria, numerical simulations were performed using plausible reproductive numbers ranging from 1.2 to 1.8 and with mean serial intervals (MSI) ranging from 2 to 4 days, consistent with previous estimates [12], [16], [20]–[23]. The length of time needed to obtain similar case notifications to the number observed around 29 May was determined. For statistical robustness 1,000 simulations for each R and MSI pair were performed and the average length and the standard deviation calculated (Table 1). For a representative intermediate estimate of R = 1.4 and an intermediate value of the MSI = 2.8 days, the average length of time to obtain the actual case numbers reported on 29 May is 33 days. This is contrasted with the actual time from the first reported case in Victoria of just 9 days.

**Table 1:** Sensitivity analysis of the effective reproduction number [R] and the mean serial interval [MSI] for the simulation results.

R\MSI	2.0	2.5	2.8	3.0	3.5	4.0
**1.2**	32.3 (9.5)	46.5 (11.5)	56.3 (14.3)	62.6 (15.5)	78.3 (20.3)	96.5 (25.2)
**1.4**	19.3 (4.9)	27.41 (6.5)	33.0 (7.7)	35.4 (9.0)	45.0 (9.7)	55.4 (13.2)
**1.5**	16.6 (4.2)	23.3 (5.5)	27.8 (6.3)	30.6 (7.1)	37.8 (7.7)	46.1 (9.9)
**1.6**	14.6 (3.6)	19.9 (5.1)	23.9 (5.5)	27.2 (5.8)	32.9 (7.0)	39.2 (8.3)
**1.8**	11.6 (2.8)	16.1 (3.7)	19.3 (4.6)	21.2 (4.8)	25.7 (6.0)	31.6 (6.4)

The table shows the simulated average number of days before 29 May 2009 that the outbreak in Victoria should have commenced, in order to match the actual case numbers observed on 29 May 2009. Each reproduction number/serial interval combination is the average number of days (and standard deviation) of 1,000 simulations for those values.

Figure 2 shows a typical example of one of the simulation results with an inputted value of R = 1.4 and MSI = 2.8 days and the reported daily number of cases. Also shown are the calculated effective reproduction numbers for the simulated and real data. The deviation away from 1.4 early in the simulated data is due to the low number of cases. Over this range of reproductive numbers and mean serial intervals the pandemic commencement date in Victoria is postulated to be somewhere between 14 April (R = 1.4, MSI = 3.5) and 9 May (R = 1.6, MSI = 2.5).

### Pandemic cases in Western Australia

The first confirmed case of pandemic influenza was notified on 24 May, four days after notification of the first Victorian case, in a traveller returning from Canada via the US. Only 23 additional cases had been notified more than two weeks later, with five linked to travel from North America and the remainder in travellers from Victoria or linked directly to Victorian-origin cases. Of the first 102 cases notified in WA, 53% were imported from Victoria or linked directly to Victorian-origin cases.

**Figure 3:**
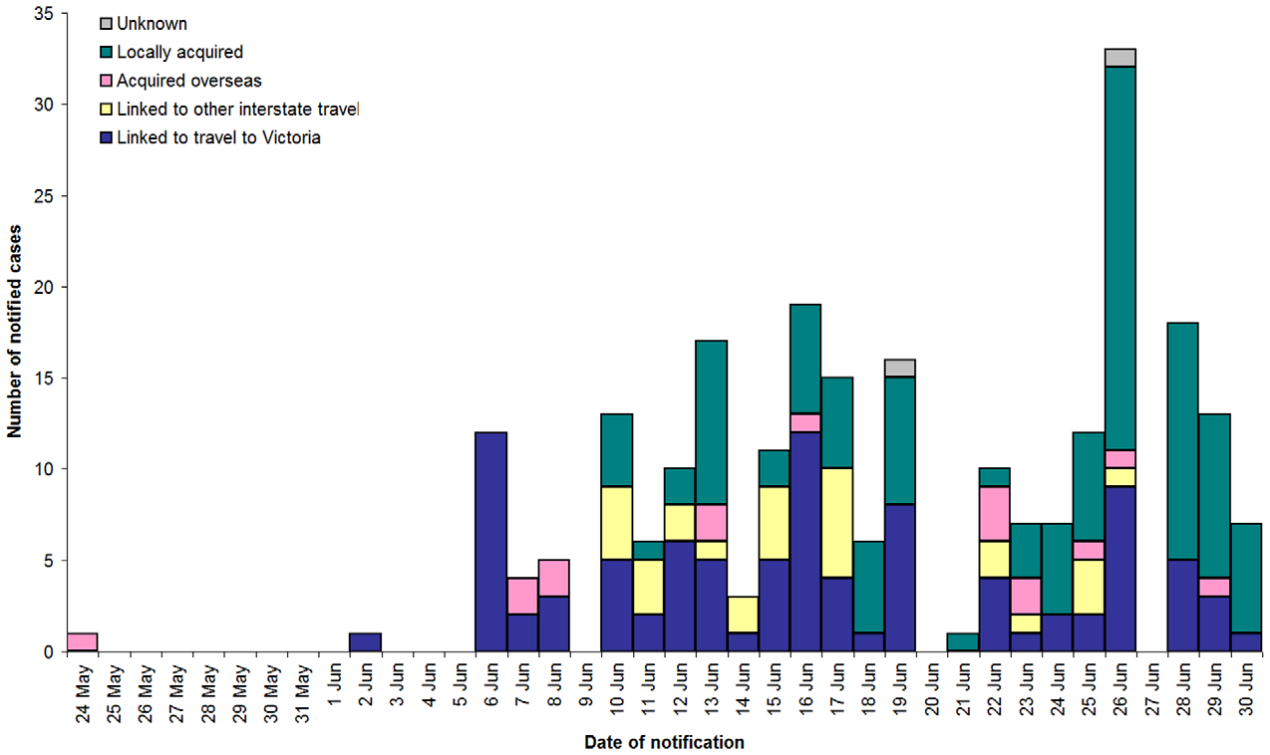
Notified cases by onset date and importation source, Western Australia 2009.

By 30 June, 247 cases had been notified in WA (Figure 3). Of these 16 (6%) were travellers from overseas countries with documented transmission, 94 (38%) were travellers from Victoria or locally acquired cases linked to Victorian-origin cases, 29 (12%) were associated with travel from other Australian jurisdictions, 106 (43%) were locally acquired with no travel history and no identifiable links to imported cases and two (0.8%) were lost to follow up. Amongst the 94 clearly documented Victorian-associated cases, there were 72 individual importations over this period, demonstrating repeated seeding of WA by persons infected in Victoria.

### Estimation of the effective reproduction number for pH1N1 influenza in Western Australia

**Figure 4:**
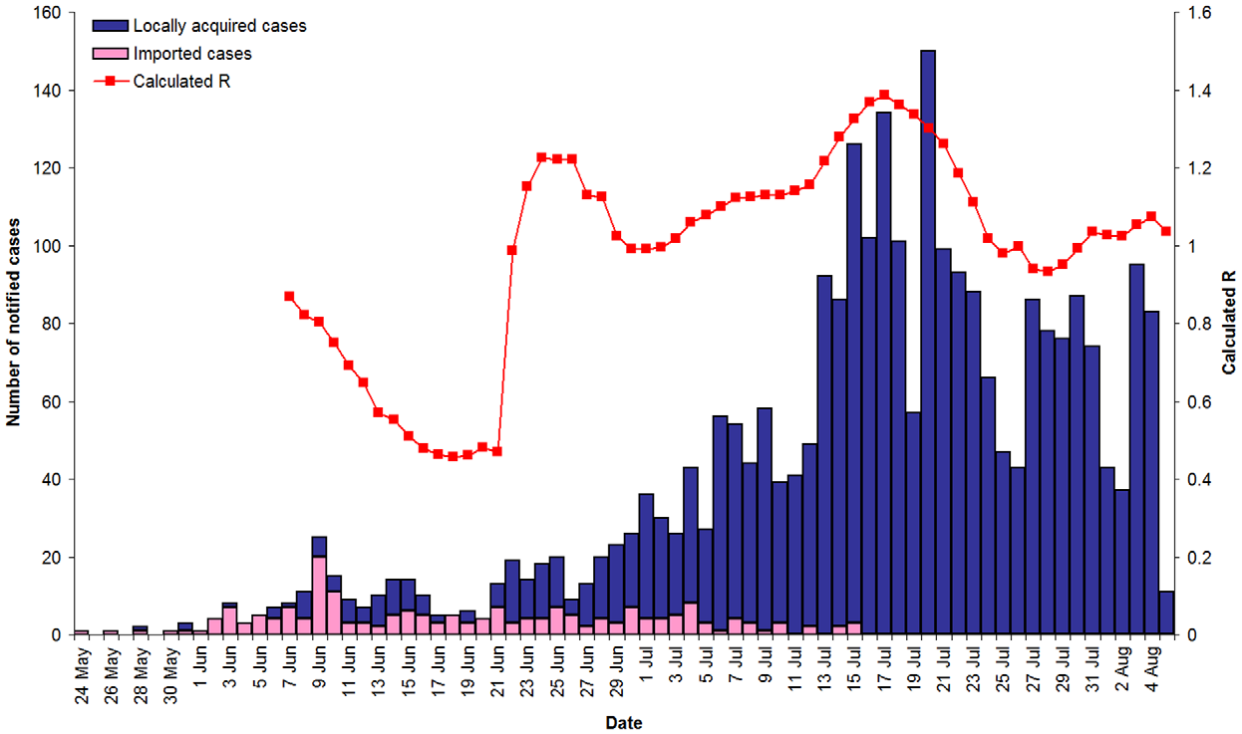
Case notification and estimated reproductive number by date using a serial interval of 2.8 days, Western Australia 2009.

Utilising the same method as used for estimating R in Victoria, and allowing for imported cases, R in WA was estimated to be well below 1 until 23 June. This suggests there was no sustained local transmission up to that date and that imported cases out-numbered local cases. From 24 June onwards the calculated values for R ranged between 1 and 1.4, with an average value of 1.2, and declined almost to 1.0 in the latter part of July, which corresponded with the peak of pH1N1 notifications in WA (Figure 4).

## Discussion

We have shown that epidemic curves and estimates for the effective reproduction number confirm the very different nature of the pH1N1 outbreaks in Victoria and WA. There appeared to be no pandemic virus in WA prior to multiple importations from Victoria, other Australian jurisdictions and overseas. Estimates of R in WA were consistently around 1.2 and never above 1.4. By contrast, initial estimates of R in Victoria approached 3, followed by a rapid – and implausible - decrease over a very short period of time. This precipitous fall cannot be explained satisfactorily by potential reduced transmission associated with early control interventions (such as partial school closure, t isolation and treatment of cases, and chemoprophylaxis and home quarantine of contacts), or depletion of susceptible people within the population. For instance, it took 20 days for the estimated value of R for the 1918–19 pandemic in San Francisco to fall from ∼2.4 to ∼1.2 [9]. We suggest the high initial value of R = 2.7 in the Victorian pandemic and the dramatic decline in 9 days is more likely explained by unrecognised cases due to an earlier commencement of the epidemic in Victoria.

The reproduction number for pH1N1 has been calculated for a number of different countries and ranges from 1.2–1.7 in Mexico, the US and Peru [20]–[23]. Higher values of R of ∼2 have been reported for Japan [16] and New Zealand [12] but these are acknowledged to be influenced by social clustering and increased diagnostic testing, and are hence most likely over-estimates of the true value of R. The estimates of R in Victoria and WA reported here have accounted for imported cases.

When the Victorian pandemic was simulated to reflect a plausible value of R = 1.4 and a serial interval of 2.8 days, earlier undetected cases needed to be invoked to reflect the observed epidemic pattern. In this scenario, modelling suggests that community transmission of the pandemic virus was most likely established by 25 April, around the time the virus was first recognised in the US and Mexico, two weeks before the first reported case in a traveller to Australia, and almost six weeks before community transmission was recognised in Victoria. Had simulations used higher values for R and lower values for the serial interval, the Victorian pandemic would have been modelled to commence even earlier. Conversely, there was no need to evoke undetected cases in WA in order to estimate a plausible range of values for R.

There was a marked difference in the proportion of imported cases in Victoria and WA. In WA 50 (49%) of the first 102 cases had travelled (eight overseas, 38 to Victoria and four to other Australian states) and a further 20 were directly linked to those cases that had travelled interstate. This is similar to the experiences of countries in the northern hemisphere. For example, in Spain 78% of the first 98 cases had acquired their infection abroad [26]; in the United Kingdom 44% of the first 65 cases reported travel to the United States or Mexico [27]; in Germany 47% of the first 198 cases were described as imported [28] and in Turkey 77% of the first 111 confirmed cases in the Turkish community were imported [29]. In Ireland 84% of the first 156 cases were imported, 14 (9%) were infected in Ireland by an imported case and two (1%) were infected in Ireland without any identifiable travel association [30].

We have previously highlighted three observations to support our hypothesis of early community transmission of pH1N1 in Victoria [3]. We have now elaborated on the first of these observations, that a low proportion of Victorian cases had any travel history or link to travellers. Travel history and exposure were collected for all 977 cases reported before commencement of the *Modified Sustain* phase, so that no cases with a travel history or exposure to travellers should have been missed.

Secondly, there was a rapid rise in the number of notifications of locally acquired cases. In Peru a period of almost five weeks elapsed from identification of the first imported case before a dramatic increase in cases was recorded [31]. This rapid rise in Victoria occurred almost immediately and could not have been a consequence of exposure to the five documented imported cases, given that all these cases were isolated and their household contacts quarantined. Either transmission was already well established in Victoria by this time, or there were continuing undetected imported cases that fuelled the epidemic. The latter is unlikely, given widespread media attention and active case-finding at that time that targeted travellers reporting influenza-like illness.

Thirdly we noted the difference in the median age of 15 years in the first 977 cases to the median age of 21 years in patients notified through the general practice surveillance scheme [32], and suggested this implied an amplification of an established epidemic in school-aged children. We have now supported these three arguments with modelling of the Victorian and WA notification data and two further circumstantial observations related to Victoria, namely, export of a case to China and an outbreak on a cruise ship in which the index case was a Melbourne resident.

Other observations also support the hypothesis of early community transmission in Victoria. We have previously established thresholds for the surveillance of influenza-like illness (ILI) in the state [33]. Normally ILI levels are below the baseline threshold when surveillance commences but, in 2009, ILI levels were above this threshold when surveillance commenced at the end of April [34]. None of the first 112 patients admitted with pH1N1 to seven hospitals in Melbourne had acquired their infection overseas [35]. Finally, it was possible to identify a presumed infectious source for only 3.7% of the first 1000 cases in Victoria (James Fielding, unpublished data).

Sub-typing of influenza A specimens archived at the Victorian Infectious Diseases Reference Laboratory between January and April 2009 did not identify any pandemic influenza viruses. We assume that mild disease would generally not have resulted in presentation for medical care and that retrospective identification of cases will be difficult.

### A plausible scenario

The first alarm about infection with pH1N1 was related to increased rates of hospitalisation and death due to severe pneumonia in young adults in Mexico [36]. Identification of pH1N1 virus from Mexican patients was in response to this concern. However, the identification of the virus in the US at around the same time was serendipitous, following the identification of two influenza A viruses, one from a study on a point-of-care test and the other from a routine surveillance system, that could not be sub-typed [2]. Concern rose in the US when it was realised that the Mexican and US viruses were essentially identical [2].

The most likely explanation for the discrepancy between the way the novel virus was detected in Mexico and the US is that the virus had been circulating far longer in Mexico than the US. One phylogenetic analysis suggests that the pandemic virus may have entered the human population between November 2008 and March 2009 [20] while a second study suggests the virus may have been causing human infections as early as September 2008 [37]. Widespread unrecognised community transmission causing mild infections may have been occurring in Mexico for some weeks or months, eventually leading to recognition of a cluster of severe pneumonia in a sub-group of susceptible young adults in April 2009. This cluster would have represented the apex of the infectious pyramid.

Indeed, identification of the pandemic virus as the cause of respiratory illness in a 6-month old Mexican infant with disease onset on 24 February has been informally reported, confirming at least two months of virus circulation in Mexico prior to recognition of the outbreak [38]. It is similarly conceivable that pH1N1 was circulating unrecognised in Victoria for several weeks before it was first detected. In those weeks specific testing was targeted at incoming travellers from North America, with no hint that the virus was already circulating in the Victorian community.

A clinical attack rate below 1.4% due to pH1N1 has been estimated for the spring of 2009 in the US [39], a clinical attack rate of 7.5% has been estimated in New Zealand for the entire influenza season between April and August 2009 [40] and in England the estimated clinical attack rate was 10 times lower than the cumulative incidence of infection of 20% suggested from serosurveys of 15–24 year olds [41]. We suggest that a relatively low clinical attack rate - but a much higher infection rate - by a virus causing generally mild disease would allow community transmission of the virus to go unrecognised for many weeks. We further suggest that this occurred in Mexico and Victoria and may indeed have occurred in other countries [42].
